# Ciencia de datos aplicada a la obtención de estimados de variación biológica

**DOI:** 10.1515/almed-2024-0163

**Published:** 2025-04-03

**Authors:** Fernando Marques-Garcia, Ana Nieto-Librero, Nerea Gonzalez-García, Xavier Tejedor-Ganduxé, Cristina Martinez-Bravo

**Affiliations:** Servicio de Análisis Clínicos y Bioquímica Clínica, Laboratorio Clínico de la Metropolitana Nord (LCMN), Hospital Universitario Germans Trias i Pujol, Badalona, Barcelona, España; Departamento de Estadística, Facultad de Medicina, Universidad de Salamanca, Salamanca, España

**Keywords:** ciencia de datos, estimados, variación biológica

## Abstract

**Introducción:**

Bajo el término ciencia de datos se agrupan una serie de herramientas y procesos que nos permiten obtener nueva información a partir de bases de datos, que pueden ser tanto estructuradas como no estructuradas. Este concepto está tomando cada vez más relevancia en el entorno sanitario. En el Laboratorio Clínico, dentro de las múltiples aplicaciones que puede tener, se han desarrollado algoritmos para la obtención de intervalos de referencia poblacionales o estimados de variación biológica (VB), entre otros. Estos algoritmos nos pueden permitir salvar las limitaciones que presentan los métodos clásicos o directos.

**Contenido:**

Revisión del estado del arte para el diseño de algoritmos encaminados a la obtención de estimados de VB, utilizando herramientas *Real-World Data* (RWD) en el entorno de la ciencia de datos.

**Resumen:**

Descripción de la estructura de algoritmos para calcular estimados de VB en base a la evidencia científica disponible. Se discuten las ventajas sobre los métodos directos, así como las limitaciones que actualmente presentan.

**Perspectiva:**

El RWD aplicado a la obtención de estimados de VB se trata de un campo novedoso, el cual ofrece un elevado potencial de desarrollo para incrementar el conocimiento sobre la VB.

## Introducción

El concepto Big Data está relacionado con la utilización de grandes bases de datos que no se pueden analizar mediante procesos estadísticos convencionales [1]. Así, basándonos en la definición del Medical Subject Headings (MeSH) del año 2024, Big Data se define como: “cantidades extremadamente grandes de datos que requieren análisis computacionales rápidos y a menudo complejos para revelar patrones, tendencias y asociaciones, relacionados con diversas facetas de entidades humanas y no humanas” (https://www.nlm.nih.gov/mesh/meshhome.html). El Big Data presenta una serie de características definidas como las “Vs”. De las múltiples “Vs” [2] tres han sido utilizadas ampliamente: volumen, velocidad y variedad [3]. De esta manera, las bases de datos poblacionales generadas en los laboratorios clínicos presentan un elevado volumen de datos, lo cual contribuye a la disminución de la incertidumbre y, por lo tanto, al aumento de la precisión de los modelos estadísticos utilizados. Estas bases se producen rápidamente, gracias al alto número de datos generados y acumulados cada día, y también se caracterizan por su variedad, ya que suelen tener un grado de heterogeneidad que permite estudiar la población mayoritaria, así como grupos más minoritarios, siempre que, en estos últimos casos, incrementemos suficientemente el número de datos.

Por otra parte, el conjunto de herramientas utilizadas para analizar los datos en el marco del Big Data se denomina Data mining. Éste se define por la MeSH (2024) como: “el uso de herramientas de análisis sofisticadas para clasificar, organizar, examinar y combinar grandes conjuntos de información” (https://www.nlm.nih.gov/mesh/meshhome.html). Recientemente se ha desarrollado el concepto de Datos del Mundo Real o *Real-World Data* (RWD) para referirse a este tipo de estudios [4], 5]. El término RWD lo define la Food and Drugs Administration (FDA) como: “datos relacionados con el estado de salud del paciente y/o la prestación de atención médica recopilados de forma rutinaria de una variedad de fuentes. Los ejemplos de RWD incluyen datos derivados de registros médicos electrónicos, datos de reclamaciones médicas, datos de registros de productos o enfermedades y datos recopilados de otras fuentes (como tecnologías de salud digitales) que pueden informar sobre el estado de salud” (https://www.nlm.nih.gov/mesh/meshhome.html). Toda esta terminología se agrupa bajo el paraguas de la ciencia de datos, la cual ha ido ganando terreno en la práctica clínica y se estima que irá en aumento en los próximos años [6]. Ciencia de datos se define por la MeSH como: “un campo interdisciplinar que implica procesos, teorías, conceptos, herramientas y tecnologías; y permite revisar, analizar y extraer un conocimiento y una información valiosa, a partir de datos estructurados y no estructurados” (https://www.nlm.nih.gov/mesh/meshhome.html).

Los sistemas informáticos de laboratorio (SIL) almacenan una gran cantidad de datos, que originalmente se solicitaron para dar respuesta a una necesidad asistencial encaminada al diagnóstico, monitorización o seguimiento de diferentes patologías. Entre estos datos se encuentran los resultados analíticos de las diferentes pruebas realizadas en los laboratorios clínicos, y otra información como datos demográficos (sexo, edad), información clínica, etc. Todos estos datos, que inicialmente se generaron para responder a una pregunta clínica, se pueden reutilizar para obtener información que permanece oculta en los mismos y que nos permitirá responder a preguntas que nos planteemos en el ámbito de la ciencia de datos. Uno de los puntos fuertes del SIL es que la información se almacena de manera estructurada (total o parcialmente), por lo que el acceso y la explotación de los datos almacenados son objetivos alcanzables.

Los estudios clínicos clásicos se han planteado de manera directa o prospectiva, en la mayoría de los casos. En el marco del laboratorio clínico, el cálculo de intervalos de referencia poblacionales (IRp) [7] o la obtención de estimados de variación biológica (VB) [8] se han desarrollado utilizando estos métodos. Con la incorporación de la ciencia de datos se produce un cambio de perspectiva, permitiendo realizar estudios de manera indirecta o retrospectiva. Así, se han llevado a cabo estrategias centradas en la utilización de grandes bases de datos para, mediante procedimientos estadísticos, obtener IRp [9], 10], estimados de VB [11] o valorar el control de calidad interno utilizando datos de pacientes [12], entre otros.

En esta revisión, nos vamos a centrar en la obtención de estimados de VB mediante la utilización de bases de datos almacenadas en los SIL y obtenidas en el campo de la ciencia de datos. Esta aproximación se plantea como una forma novedosa para la obtención de estos estimados de VB [13].

## Estimados de variación biológica

En condición de equilibrio estable (*steady state*) la concentración de los mensurandos presenta, por una parte, variaciones predecibles o sistemáticas como son los ritmos circadianos, y, por otra parte, variaciones aleatorias medibles como la variación biológica (VB) [14].

La VB se define como la fluctuación de los mensurandos alrededor de su punto de homeostasis [15]. Ésta se divide en dos componentes: VB intra-individual (CV_I_) y VB inter-individual (CV_G_).

La VB intra-individual (CV_I_) representa la variación de la concentración/actividad del mensurando alrededor del punto de control homeostático dentro de cada individuo [13]. Sin embargo, la VB inter-individual (CV_G_) determina la variación entre los puntos homeostáticos de control de diferentes individuos de la población [13].

Los valores de estos estimados pueden variar dependiendo de la homeóstasis de cada magnitud [16], así como de otros factores como, por ejemplo: la edad, el sexo o la condición fisiopatológica del individuo [17].

La primera base de datos en la que se recogían estos estimados de VB fue elaborada por Ricos et al. [18]. En ella se incluían estimados para 316 mensurandos obtenidos de diferentes fuentes bibliográficas (Base histórica-SEQC^ML^). Recientemente, en el año 2019, se publicó la nueva base de datos (Base de datos actual) elaborada por el Grupo de Trabajo de la Base de Datos de Variación Biológica (TG-BVD) de la Federación Europea de Química Clínica y Medicina de Laboratorio (EFLM) en colaboración con el Grupo de Trabajo sobre Variación Biológica (WG-BV) [19]. En esta base, la calidad de los artículos se ha valorado siguiendo el Listado de Revisión Crítica de Datos de Variación Biológica (BIVAC) y el estimado global se ha derivado de un *meta*-análisis en base a la categorización utilizando los criterios marcados por el BIVAC (grado A, B, C o D) [20].

Los estimados de VB presentan diferentes aplicaciones en el laboratorio clínico como son: el establecimiento de especificaciones de rendimiento analítico (APS) [21]*,* la estimación del punto de control homeostático individual (HSP) [16], el cálculo del valor de referencia del cambio (VRC) [13], la obtención del índice de individualidad (II) [22] y el establecimiento de intervalos de referencia personalizados (IRper) [23].

## Características de los métodos directos

Los métodos directos para la obtención de estimados de VB son los más ampliamente utilizados, especialmente el método de Fraser y Harris [24]. Estos métodos se caracterizan por el control estricto de los individuos incluidos en el estudio (“individuos normales”), utilizando para ello encuestas de salud y la valoración analítica de cada uno de los participantes [25]. La definición de población “normal” siempre se ha asimilado a población sana, pero el concepto de salud “es una condición relativa carente de una definición universal”, no existiendo en la actualidad una estandarización de la definición de la misma [26]. Manrai et al. [26] describen los resultados de una encuesta realizada en el año 2013–2014 por el US Centers for Disease Control and Prevention´s National Health and Nutrition Examination Survey (NHANES) en la que se presentan tres definiciones de salud: ausencia de enfermedades comunes, individuos entre 18 y 40 años y autovaloración subjetiva de buen estado de salud. Únicamente el 5 % de los encuestados cumplía los tres criterios a la vez. Esto marca la dificultad de seleccionar una población normal *a priori.* Además, el método de Fraser y Harris se caracteriza por tres premisas fundamentales: asume que los datos de VB deben seguir una distribución normal, con homocedasticidad de las varianzas y sin tendencias en los datos (población en equilibrio estable). En este método, las muestras siempre se han de analizar en duplicado, utilizando como procedimiento estadístico para la obtención de los estimados el método de análisis de la varianza (ANOVA) anidado [20].

## Estudios publicados con estimados de variación biológica por métodos indirectos

La utilización de estudios indirectos o estrategias de RWD para la obtención de estimados de VB ha comenzado a aplicarse hace relativamente pocos años. A día de hoy, solamente se han publicado cinco artículos en los que se describa la obtención de estimados de VB por estrategias RWD, lo cual representa un nivel de evidencia científica limitada. Los artículos publicados han sido: Loh et al., quienes obtuvieron estimados de VB en individuos pediátricos [27], Jones, quien obtuvo estimados en individuos adultos para diferentes parámetros bioquímicos y hormonas (sexuales y adrenales) [28], 29] y Marqués-Garcia et al., quienes desarrollaron un nuevo modelo para la obtención de estimados de VB mediante estrategias RWD encaminado a aumentar la robustez de los métodos publicados hasta ese momento [11]. En estos cuatro artículos se publicaban solamente estimados del componente CV_I_ de las magnitudes estudiadas. Por su parte, Loh et al. publicaron otro artículo en el que obtuvieron estimados CV_G_ utilizando la estrategia RWD [30].

## Características de los estudios RWD para la obtención de estimados de variación biológica

Los métodos indirectos basados en RWD reutilizan datos que se han generado para el diagnóstico y seguimiento de los individuos tratando de identificar nueva información, como los estimados de VB en este caso [9]. Estos métodos se presentan como una alternativa a los métodos directos como el de Fraser y Harris [24]. La definición de la base de datos con la que se va a trabajar es el primer paso a tener en cuenta antes de comenzar el estudio estadístico. En primer lugar, se ha de seleccionar la población a incluir en la base de datos, siendo la de pacientes de atención primaria la que se presenta como más estable para este tipo de estudios. En función del análisis que se realice puede ser necesario obtener bases de datos de pacientes ingresados y/o procedentes de consultas externas. Este es el caso de la obtención de estimados de VB en grupos de individuos patológicos portadores de una patología concreta. La aplicabilidad de métodos directos en enfermos es más complicada, dada la fragilidad que presentan estos grupos. Por tanto, disponer de estimados de VB en enfermos sería de gran utilidad porque se obtendrían estimados específicos, evitando el uso de estimados de individuos sanos para el manejo de individuos enfermos [31]. Cuanto más variable sea la base de datos, mejores conclusiones podremos obtener de los estudios basados en RWD. Así, la inclusión de más de un hospital permite ver el efecto que puede tener, por ejemplo, la distribución geográfica de los individuos (como realizan Marqués-García et al. en el que incluyen tres hospitales [11]) o la plataforma analítica utilizada. Además, utilizar periodos largos (mínimo de 12 a 18 meses) facilita el acceso a la recogida de un mayor número de fuentes de variabilidad dentro del estudio [11]. El número de individuos que forma parte del análisis no suele ser un obstáculo a tener en cuenta, dado el gran tamaño de las bases de datos utilizadas. Si se toma como referencia los modelos RWD para IRp es necesario disponer como mínimo de 10.000 individuos en la población total [10] y 400 individuos por cada subgrupo realizado de la población total [32]. La posibilidad de dividir la base de datos en subgrupos representa una fortaleza muy importante de los métodos basados en RWD. Así, esta población total se puede subdividir en base a diferentes criterios como son edad o sexo, entre otros. Sin embargo, los métodos directos al utilizar un número reducido de individuos no permiten realizar este tipo de agrupaciones. Un aspecto importante de estos estudios, dado que se recogen resultados de largos periodos de tiempo, es garantizar la estabilidad de los mismos mediante el aseguramiento de la calidad apoyándonos en la evaluación de los resultados obtenidos del control de la calidad interno y los programas de control externo de la calidad.

La base de datos obtenida se ha de limpiar para evitar que los valores atípicos (outliers) interfieran en el resultado de los estimados de VB. A pesar de fijar criterios de inclusión/exclusión durante el proceso de obtención de la base de datos no podemos asegurar que todos los individuos sean sanos [9]. Por esta razón, es necesario detectar y eliminar los valores potencialmente patológicos y seleccionar aquellos que corresponden a los individuos sanos. Así, se pueden utilizar diferentes aproximaciones estadísticas para la eliminación de outliers, como el método de Tukey [27], 30], o aproximaciones con un componente más biológico, como es el valor de referencia del cambio (VRC) [11]. De este modo, Loh et al. utilizan el método de Tukey para la eliminación de los valores atípicos [27], 30]. Por otro lado, Marqués-García et al. comparan el método de eliminación mediante la aplicación del rango intercuartilíco frente a la aplicación del VRC, concluyendo que el VRC representa una herramienta más útil para la eliminación de valores atípicos por presentar un componente biológico en la ecuación de cálculo [11]. Otra posibilidad es no eliminar valores atípicos y utilizar métodos estadísticos para tratar de aislar la población de datos de individuos sanos de la población de datos de individuos enfermos, como, por ejemplo, puede ser la utilización del método de Bhattacharya [33], que realiza Jones et al. en sus dos trabajos publicados [28], 29].

Finalmente, con la base de datos filtrada, se aplican métodos estadísticos que permitan obtener los estimados de VB. Los métodos estadísticos se pueden agrupar en métodos paramétricos y métodos no paramétricos. De esta forma, se han utilizado aproximaciones paramétricas para describir la distribución central de los datos y estrategias no paramétricas, utilizando la técnica de regresión no lineal basada en splines cúbicos suavizados o mediante técnicas de remuestreo o bootstrap no paramétrico. Así, Jones et al. utilizan la aproximación paramétrica obteniendo el valor del CV_T_ en base al método Bhattacharya, y calculando el estimado CV_I_ como la diferencia entre el valor CV_T_ y el coeficiente de variación analítico (CV_A_). Por su parte, Loh et al. [27], 30] y Marqués-García et al. [11] utilizan las aproximaciones no paramétricas. Ambos métodos calculan el valor del CV_I_ individual como la diferencia entre el valor del CV_T_ y el valor del CV_A_. La diferencia entre los dos radica en la estrategia para la obtención del estimado CV_I_ global de la población. Loh et al. utiliza los splines cúbicos suavizados obteniendo el CV_I_ global como la mediana de los CV_I_ individuales y en cambio Marqués-García et al. utilizan el método de remuestreo o bootstrap no paramétrico en el cual se realiza un remuestreo en grupos de datos de igual tamaño calculando la mediana del CV_I_ en estos grupos.

A día de hoy, no hay un consenso sobre cuál es el algoritmo más idóneo de los tres publicados. Pero a pesar de esto, el trabajo de Marqués-García et al. [11] corrige muchas de las limitaciones publicadas en los trabajos previos. Por ejemplo: se trata de un estudio multicéntrico, se incluye un mayor volumen de datos en el estudio, se eliminan los valores atípicos mediante la aplicación de una estrategia con un fundamento más biológico y menos estadístico (como es el VRC), se obtienen estimados con buena correlación con la base de datos de variación biológica de la EFLM [19], y, al utilizar la metodología de bootstrap para la obtención del estimado CV_I_ global, se permite calcular el intervalo de confianza del estimado. Loh et al. publicaron el único artículo en el que obtuvieron estimados CV_G_ utilizando la estrategia RWD [30]. Para ello calcularon este estimado mediante la aplicación de la ecuación propuesta por Fraser y Harris [24] para la estimación del CV_G,_ pero modificada para grandes volúmenes de datos.

## Ventajas y limitaciones de los estudios RWD para la obtención de estimados de variación biológica

El desarrollo de algoritmos centrados en el análisis de bases de datos para la obtención de estimados de VB permite una aproximación diferente a la de los métodos directos, tratando de mejorar las limitaciones que éstos presentan. Así, con los métodos RWD, se puede valorar la variabilidad presente en la base de datos utilizando resultados obtenidos en condiciones reales de trabajo y no bajo condiciones idealizadas, como sucede en los métodos directos. Además, los métodos RWD son menos invasivos, ya que se re-utilizan datos, y no es necesario realizar extracciones periódicas como sucede en los métodos directos. Por su parte, los métodos directos son más costosos que los métodos RWD al necesitarse material de extracción de muestras, reactivos, instalaciones, etc., específicos para el estudio. Otro aspecto a tener en cuenta en los estudios directos es el concepto de normalidad. Al realizarse con un número de individuos pequeño puede tener cierto efecto la presencia de patologías subclínicas en el valor de los estimados de VB obtenidos. En cambio, cuando se utilizan grandes bases de datos analizadas mediante RWD este efecto se puede ver minimizado. Asimismo, los valores de los estimados determinados por métodos RWD presentan una buena correlación con los obtenidos por métodos directos [11], demostrando la potencia de estos algoritmos para separar la señal (VB) del ruido de los datos.

Las estrategias RWD presentan limitaciones que será necesario ir superando. A día de hoy, aún no hay una normalización en relación con las historias clínicas [34]. Se está comenzando a trabajar en esta línea, aunque esta falta de normalización no solamente sucede entre países, si no, también, dentro de cada país. Así, disponer de programas de historia clínica electrónica comunes entre los diferentes territorios permitiría una explotación homogénea de los datos, tanto en la estructura de las bases de datos que se obtendrían como en la estandarización de la nomenclatura adoptando sistemas de codificación internacionales como el sistema LOINC o SNOMED-CT. Alcanzar los mejores estándares de calidad en el grado de normalización repercutirá en la calidad de los datos que están disponibles. En este sentido, otra limitación es la protección de datos. Se debe trabajar en mejorar la capacidad de anonimización de estos datos para salvaguardar la información de cada paciente [35]. Del mismo modo, una posible estrategia de trabajo sería la generación de redes seguras entre hospitales que, además de la anonimización de los datos, permitan la circulación de los mismos en entornos seguros. Con respecto a las herramientas estadísticas se utilizan procedimientos complejos que requieren de un conocimiento avanzado, así como de disponer de programas específicos para estos cálculos. En el caso de la obtención de estimados de VB por métodos RWD el nivel de evidencia científica es aún bajo al disponerse solamente de cinco artículos publicados. De esta manera resulta indispensable seguir trabajando en los algoritmos que permiten calcular los estimados de VB para aumentar la robustez de los valores obtenidos. Otros aspectos a mejorar se basan en la estabilidad de los datos del estudio (datos en condición de equilibrio estable), condición que aún no se detalla en estos estudios. También sería interesante tratar de aumentar el periodo de recogida de datos para tener en cuenta la mayor cantidad posible de fuentes de variabilidad.

## Conclusiones

La utilización de estrategias Real-World Data (RWD) se está desarrollando para llevar a cabo estudios en el entorno de los sistemas sanitarios. En el laboratorio clínico se aplica desde hace unos años para la obtención de intervalos de referencia poblacionales, aunque recientemente se han desarrollado nuevas aplicaciones como son el control de calidad interno basado en medias de pacientes y en la obtención de estimados de VB. La aproximación RWD para la obtención de estimados de VB muestra notables ventajas en comparación con los métodos clásicos basados en aproximaciones directas. Por ejemplo, son más baratos y menos laboriosos, a pesar de que el manejo de los datos y el tratamiento estadístico es más complejo que en los métodos directos. Aunque ya hay un artículo multicéntrico publicado, añadir más centros hospitalarios en estos estudios nos permitiría analizar las diferencias en los valores de los estimados de VB por área geográfica o entre las plataformas analíticas que se puedan incluir. Al tratarse de grandes volúmenes de datos se dispone de una gran cantidad de información que permite segregar la población en diferentes subgrupos, obteniendo estimados de VB específicos para cada uno de ellos. Así, se pueden obtener estimados en grupos vulnerables como pediátricos, ancianos o enfermos y, de este modo, disponer de estimados específicos para realizar un manejo más individualizado de los pacientes. A pesar de los datos ya disponibles de estimados de VB por métodos RWD, es necesario continuar con en el diseño de algoritmos de trabajo que posibiliten la obtención de estimados de VB robustos para poder aplicarlos con la mayor precisión posible, e ir acumulando evidencia científica con el objeto de conseguir los mejores algoritmos de trabajo posibles. Los resultados obtenidos en estos estudios se han de validar para valorar su idoneidad como, por ejemplo, comparándolos con el estándar oro actual correspondiente a la base de datos de VB de la EFLM [19]. Actualmente, el Proyecto Multicéntrico Español (BiVaBiDa) está trabajando activamente en el desarrollo de aplicaciones para la obtención de estimados de VB mediante RWD en diferentes grupos de interés.
